# Improving patient compliance with diabetic retinopathy screening and treatment

**Published:** 2015

**Authors:** Karinya Lewis

**Affiliations:** Specialist Registrar, University Hospitals Southampton, Southampton, UK. **Karinya.lewis@uhs.nhs.uk**

**Figure:**
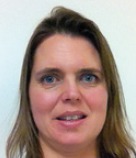
Karinya Lewis

Diabetic retinopathy is one of the many complications of diabetes. Because there are no symptoms initially, patients will not realise that they have the condition until it is at a proliferative stage or they develop macular oedema, when their vision becomes affected. Unfortunately, vision that has been lost may never be regained.

To prevent visual loss, early detection is needed at the pre-proliferative stage. This can only be achieved if the person with diabetes has regular (often annual) examination of the retina, starting from when they are first diagnosed. Screening of diabetes patients therefore has to be timely and in accordance with locally agreed guidelines for detection, referral and treatment. The challenge faced across many programmes is that people with diabetes:

- Do not always attend regular DR screening
- Present with late-stage retinopathy which results in a poor visual outcome
- Have poor acceptance of laser treatment.

In national population-based screening programmes, the desirable target uptake is 80%, which is difficult to achieve. The UK National Screening Programme took five years from the start of the programme in 2006 to reach this target. Attendance for initial laser treatment is reportedly around 70%, but in some studies as few as 21–45% of those patients who started laser treatment had completed the course of laser when they were followed up 6 months later.

## Why do patients not attend?

Reasons for non-attendance in various setting have been studied qualitatively and quantitatively and common themes arise.

### Patient-related reasons

These can be remembered using the first 7 letters of the alphabet.

- **A**wareness about diabetes and eye complications is soften limited. Patients may not be aware of local screening centres
- **B**elief that they do not require retinal examinations or treatment as their vision is good, or they have a mild form of diabetes, or are too old
- **C**ost: direct and indirect (e.g. travel)
- **D**istance from screening/treatment centres and discomfort from dilating drops
- **E**ffort to attend yet another clinic. People with diabetes often have multiple hospital appointments
- **F**ear of laser treatment and fear of its impact on quality of life and jobs. A lack of family support
- **G**uilt surrounding failure to control blood glucose levels. People fear that an eye examination, or being told they need laser treatment, will confirm their guilt and make them feel even worse.

### Provider-related reasons

- The existence of poor counselling and advisory services about ocular complications for people with diabetes
- An inefficient system for getting patients to come, and to then come back if needed (‘call and recall’ systems)
- Long waiting times for screening or treatment
- Complicated referral mechanisms or inaccessible locations where services are offered.

## Assessing the situation

The different factors in patients' experience – which either prevent or encourage their engagement – should determine what interventions might improve uptake of services. By assessing the situation and identifying key stages in the process (from screening to completion of treatment) we can target interventions at those most at risk of vision loss.

### The non-attendance rate

This is the proportion or percentage of patients who do **not** attend their appointments, whether for their yearly eye examination or for laser treatment. We should aim to make this figure as low as possible.

At a clinic level, work out the non-attendance rate, say for 1 month, by dividing the number of patients who did not attend their appointment (for screening, the eye clinic or laser treatment) by the number of patients who have appointments in that time period. Multiply by 100 to obtain the percentage.

### Coverage

Coverage is the percentage or proportion of the target population who undergo screening. In the case of diabetic eye disease, ‘screening’ means yearly eye examinations for everyone diagnosed with diabetes. Coverage is an important measure of the quality of a programme, and we should aim to make this figure as high as possible.

To work out the coverage offered by your clinic or programme, divide the number of patients who attended screening on a yearly basis by the number of patients with diabetes in your catchment population; multiply by 100 to calculate the yearly coverage of screening as a percentage.

### The pathway

Can you identify which part of the pathway, from screening to treatment, is most affected by non-attendance?

Is there a particular geographic location where assessments or treatment take place, where non-attendance is higher?

### Who is not coming?

Among the diabetes patients, can you identify any particular subgroup who would benefit most from a targeted intervention? (For example, younger patients, those newly diagnosed, people with language barriers, or people with low social economic status or poor education.)

## Addressing the challenges

The following practical suggestions are gathered from patient recommendations, models of good practice and successful interventions. Together, they improve the overall patient experience, improve ease of access to services, and encourage and engage the patient through education.

### Empower health professionals

- Encourage all allied health professionals working with diabetes to personally recommend annual retinal checks to patients with diabetes. Train health workers to offer intensive patient education programmes to all newly diagnosed patients, covering specifically diabetes, potential blindness and the eye.
- Encourage health workers to support patients with diabetes (especially those with poor control) and work with them to find solutions to the challenges of having diabetes. It is vital not to blame the patient or make them feel guilty.

### Strengthen patient communication

A diabetes ‘passport’ has been a useful tool to encourage patients to feel ownership of their disease and facilitate communication between health professionals and the patient. The patient brings the passport (a specially designed booklet or file) to every appointment and health professionals record current medications and results (blood sugar, blood pressure, cholesterol, kidney function, podiatry assessment and retinopathy grading) as well as when next assessments are due. The passport helps to start conversations with the patient about their diabetes.

**Figure:**
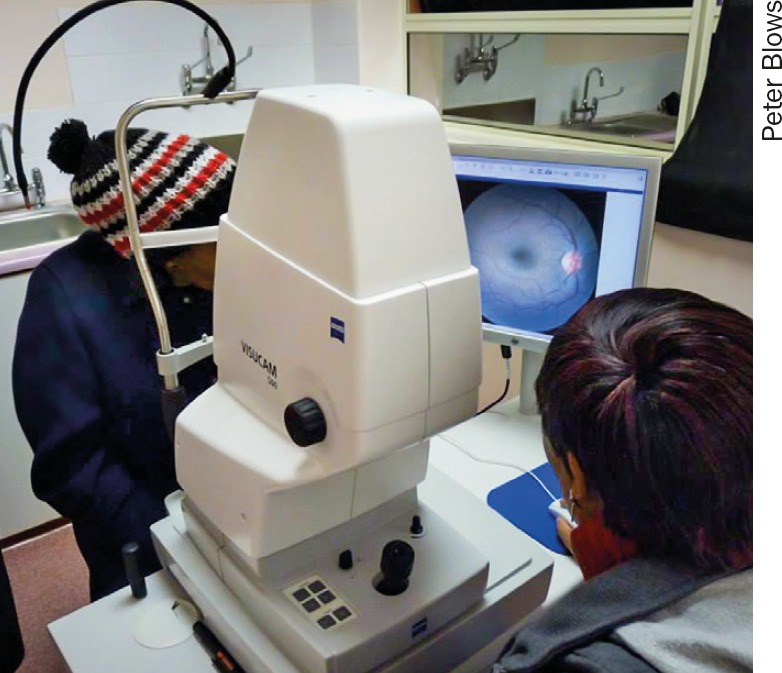
Show patients their retinal images and highlight any changes. BOTSWANA

### Offer personalised annual education

- Screeners or ophthalmologists can show patients their retinal images and highlight any changes (improvements or deteriorations) to encourage future attendance and good glycaemic control.
- Information should be available to the patient in their preferred language and in large print.

### Identify and engage patients who frequently fail to attend

- A common policy in eye clinics is to discharge patients who do not attend on two occasions. However, in diabetic eye services, these patients should be identified and contacted personally to understand their reasons for poor attendance (e.g. timing, transport, or anxiety) and solutions must be found.
- Set up a reliable system. For example, use text messaging and send reminders for patients about their appointments.
- Large-scale programmes benefit from employing a diabetic retinopathy co-ordinator who is responsible for monitoring the quality of the programme and ensuring that people keep coming back for their appointments. For more information on this, see **www.gov.uk/topic/population-screening-programmes/diabetic-eye**

## Practical considerations

Giving attention to the following practical arrangements can support patients to attend their appointments more regularly.

### Cost and accessibility

- Minimise the cost to the patient by reducing the time required and the distance travelled.
- Locate screening where there are good transport links.
- Ensure that patients can change the appointment to a more convenient time, especially if they are employed.
- Patients prefer their annual visits to be repeatable. Keep the location and routine the same, if possible, so they can become familiar with the process.

### Waiting times and dilation

- Seeing patients punctually and efficiently will reduce time off work and encourage them to return each year.
- Retinal photography without dilation drops is possible, but in older patients with cataract their photographs may be ungradeable and patients will need to be called back, unless quality assessment is done by the photographer at screening.

### Centralised services

- Some services have combined diabetic retinopathy screening with other check-ups such as blood pressure monitoring or annual flu immunisations.
- Centralised booking systems can reduce administration and costs but may offer less flexibility for those who present opportunistically, or for family members who want to attend together.

## Improving compliance with laser treatment

- Educate patients about laser treatment, its intended effect, the need to complete the course (at least two visits are usually needed) and the need to allow time to evaluate its effectiveness. This should take place at the time of consenting to laser treatment.
- Written and visual information (retinal images) supporting the discussion should be available.
- The health professional applying the laser should ensure that the patient is made comfortable, with appropriate anaesthesia and minimum effective power settings.
- If unable to achieve comfortable laser with topical anaesthesia, there should be the option to give a local anaesthetic block, or even general anaesthesia with indirect laser.
- Health professionals should understand the discomfort which may be caused by laser, and offer sympathy rather than irritation or denial, thereby establishing a good relationship and encouraging the patient to return.

## Conclusion

Improving patient engagement with preventative services requires persistent effort and innovation from service providers. Whilst laser treatment is still the best way of preventing significant visual loss, we are in a new era of treatment with anti-VEGF injections which are given monthly. Improving patient engagement, education, and compliance will be even more crucial if these new treatments are to be effective.
